# Legume-rhizobia symbiosis: Translatome analysis

**DOI:** 10.1590/1678-4685-GMB-2023-0284

**Published:** 2024-07-01

**Authors:** María Martha Sainz, Mariana Sotelo-Silveira, Carla V. Filippi, Sofía Zardo

**Affiliations:** 1 Universidad de la República, Facultad de Agronomía, Departamento de Biología Vegetal, Laboratorio de Bioquímica, Montevideo, Uruguay.

**Keywords:** Symbiosis, translatome, Poly-seq, Ribo-seq, Dual-seq

## Abstract

Leguminous plants can establish endosymbiotic relationships with nitrogen-fixing soil rhizobacteria. Bacterial infection and nodule organogenesis are two independent but highly coordinated genetic programs that are active during this interaction. These genetic programs can be regulated along all the stages of gene expression. Most of the studies, for both eukaryotes and prokaryotes, focused on the transcriptional regulation level determining the abundance of mRNAs. However, it has been demonstrated that mRNA levels only sometimes correlate with the abundance or activity of the coded proteins. For this reason, in the past two decades, interest in the role of translational control of gene expression has increased, since the subset of mRNA being actively translated outperforms the information gained only by the transcriptome. In the case of legume-rhizobia interactions, the study of the translatome still needs to be explored further. Therefore, this review aims to discuss the methodologies for analyzing polysome-associated mRNAs at the genome-scale and their contribution to studying translational control to understand the complexity of this symbiotic interaction. Moreover, the Dual RNA-seq approach is discussed for its relevance in the context of a symbiotic nodule, where intricate multi-species gene expression networks occur.

## Legume-rhizobia symbiosis

Leguminous plants like soybean can establish an endosymbiotic relationship with nitrogen-fixing soil rhizobacteria. As a consequence of this mutualistic interaction, the plant undergoes significant metabolic and nutritional changes (Oldroyd et al., 2011; Concha and Doerner, 2020) as a new root organ - the symbiotic nodule - is developed, providing an environment suitable for atmospheric nitrogen gas fixation, i.e. reduction to ammonia. This process, known as symbiotic nitrogen fixation (SNF), produces ureid or amide compounds that are exported to the rest of the plant, where nitrogen is incorporated into amino acids and other nitrogen-containing metabolites (Van Heerden et al., 2007). The plant, in turn, provides carbon and energy in the form of C4-dicarboxylic acids such as malate and succinate (Poole et al., 2018).

**Figure 1 -:**
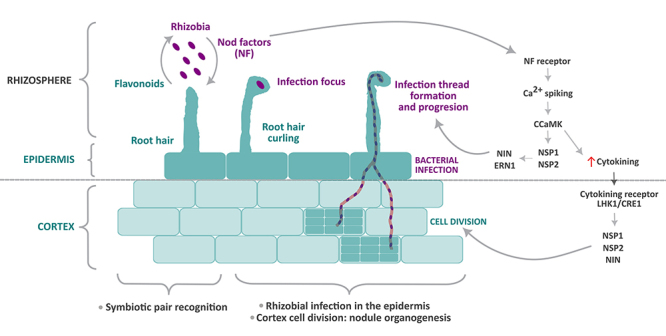
Main events occurring during the initial stages of legume-rhizobia symbiosis. The interaction initiates at the root hairs, where the exchange of molecular signals - plant flavonoids and rhizobia Nod factors (NF) - occurs. When the interaction between the symbiotic pair is effective, the rhizobia adheres to the root hair, which curls, entrapping the bacterium and forming an infectious focus. Then, an infection thread is formed due to cell wall degradation and invagination of the root hair plasma membrane. This tubular structure progresses, allowing the rhizobia to infect the root cortex, where the activation of cortical cell division originates the nodule meristem (nodule organogenesis). The two highly coordinated genetic programs activated during legume-rhizobia symbiosis are bacterial infection and cortex cell division. The first one takes place at the epidermis and involves the perception of NF through NF receptors, calcium oscillations, perception of the calcium oscillations via calcium-activated kinase (CCaMK), and transcription factors (NSP, NIN, ERN1) leading to gene expression activation. An increase in cytokinin levels promotes cortical cell division, where the signaling pathway that involves the cytokinin receptor LHK1/CRE1 and transcription factors such as NSP1/2 and NIN, among others, leads to nodule organogenesis.

The association between legumes and rhizobia is very specific; each rhizobia strain has a defined host range, which can be narrow or broad. Even though symbiotic promiscuity is widely dispersed, a rhizobia strain can only be considered effective if it can form nitrogen-fixing nodules (Perret and Staehelin, 2000). The interaction between the symbionts initiates at the rhizosphere (the region of soil that surrounds plant roots) through the exchange of molecular signals: plant flavonoids and rhizobium Nod factors, which leads to the adhesion of the bacterium to the root hair (Figure 1). Consequently, the root hair curls and entraps the bacterium, forming an infectious focus. At this point, cell wall degradation and invagination of the plasma membrane in the curled root hair result in the formation of the infection thread. This tubular structure allows the invasive rhizobia to infect the root cortex; concomitantly, activation of cortical cell division originates the nodule primordium. Then, bacteria are internalized into these nodule cells, where they become surrounded by a plant-derived membrane forming organelle-like structures called symbiosomes, where they differentiate into bacteroids - the N_2_-fixing form of rhizobia (Poole et al., 2018; Roy et al., 2020; Zanetti et al., 2020). Subsequently, nodules grow by cell division or cell expansion, forming indeterminate or determinate nodules, which develop persistent or transient meristems, respectively (Roy *et al*., 2020). 

Two independent but highly coordinated genetic programs are activated during legume-rhizobia symbiosis: bacterial infection in the epidermis and the promotion of cell division in the cortex to form the nodule meristem, i.e. nodule organogenesis (Figure 1). Epidermal cells perceive the Nod factors (lipochitooligosaccharides) through Nod factor receptors (receptor-like kinases) that activate calcium spiking via a set of proteins (symbiosis receptor-like kinases, components of the nuclear pore, and cation channels). Calcium oscillation perception involves the calcium-activated kinase CCaMK, which functions with transcription factors (such as NSP1/2, NIN and ERN1) to activate gene expression. As a result of this signaling pathway, both the initiation of bacterial infection at the epidermis and the promotion of cell division in the cortex occur. In the latter tissue, nod factors-induced cytokinin signaling involves the cytokinin receptor LHK1/CRE1, response regulators, and a multitude of transcription factors (NSP1/2, NIN, etc.) which activates a networking cascade leading to nodule organogenesis (Oldroyd et al., 2011; Tiwari et al., 2021) (Figure 1). 

Developing and maintaining nodules is resource-intensive, so the plant exerts tight control over the number of nodules forming on its roots. This way, legumes have evolved different molecular pathways, enabling them to control nodulation in response to different growing conditions. The establishment of symbiosis triggers a mechanism called autoregulation of nodulation, by which the host plant systemically controls the number of nodules to balance symbiotic nitrogen fixation with plant photosynthesis and growth (Ferguson et al., 2018). Likewise, the nitrogen regulation pathway inhibits nodulation in nitrogen-rich growing conditions, helping the plant conserve resources. Biotic and abiotic constraints are other factors that can trigger mechanisms of regulation of nodulation to help conserve plant resources under unfavorable conditions (Ferguson *et al*., 2018). In fact, SNF is a very drought-sensitive process, particularly in soybean, where even modest soil water deficits are detrimental to its productivity. 

The acquisition of nitrogen through SNF provides legume plants, a diverse group including many important food, feed, and pasture species, with a competitive advantage compared to non-legume plants (Ferguson et al., 2018). Also, the legume-rhizobia symbiosis is a key part of the nitrogen cycle in both agricultural and natural environments since it helps incorporate nitrogen into the soils and thus has important implications for both natural ecosystems and agriculture (Zanetti et al., 2020). Therefore, the importance of continuing to deepen our understanding of the various mechanisms involved in the sequential steps required to establish successful legume-rhizobia symbiosis and posterior nodulation control mechanisms is evident. Translational control arises as a candidate to help dissect those mechanisms with the aim of optimizing nitrogen fixation and improving crop productivity. 

## Why analyze the translatome?

It is well known that gene expression can be regulated along all the stages of the process: transcriptional regulation, post-transcriptional regulation, translational regulation, post-translational regulation, and protein turnover (McManus et al., 2015). Until very recently, the focus of analysis was at the level of transcriptional regulation, studying the transcriptome, determining the messenger RNAs (mRNA) abundance (Brown and Botstein, 1999; Wang et al., 2009; Krishnamurthy et al., 2018; Shulse et al., 2019). It has been demonstrated, however, that an increase in the levels of mRNA does not always correlate with an increase in the abundance or activity of the coded protein (the final point of gene expression) (Larsson et al., 2013). The translatome refers to all mRNAs recruited to ribosomes for protein synthesis. Therefore, analyzing the translatome can reveal important information about gene expression and the array of biological pathways that are active in a cell or organism and is more accurate for estimating the expression level of some genes (Kawaguchi et al., 2004; Larsson *et al*., 2013; Vogel and Marcotte, 2013). In fact, in the past two decades, the interest in the role of translational control of gene expression has increased since it has been shown in different organisms that it provides a better approximation of downstream protein abundance profiles. This way, the subset of mRNA being actively translated reflects the functional reading of the genome, outperforming the information gained when only the transcriptome (total RNA) is analyzed (Chassé et al., 2017).

The machinery for protein synthesis is highly conserved from an evolutionary point of view between plants and other eukaryotes. However, as plants are sessile organisms, specific variations in the translation machinery have occurred for adaptation to different environmental conditions, motivating the plant science community to put more effort into studying translational control in plants. Gene translational regulation is part of an ample network of RNA-level processes, including RNA quality control and turnover. The analysis of the translatome is critical for the understanding of gene expression and can be used for integration in high-throughput functional genomics screens (Urquidi Camacho et al., 2020). 

Translational control refers to regulating gene expression by controlling the levels of protein synthesized from its mRNA, which is crucial in defining the proteome, and it is used in a wide range of biological situations (in response to endogenous or exogenous stimuli), particularly relevant where transcription is silent or when local accumulation of proteins is required (Gebauer and Hentze, 2004; Hershey et al., 2012). There are two general modes of control: 1. Global control, which regulates the translation of the majority of the cell mRNAs. Many of the global translation control examples that have been described affect the translation initiation, regulating the phosphorylation or availability of initiation factors. 2. Specific control in which a specific group of mRNAs expression is regulated without affecting the translational status of the cell. The latter is driven by the recognition by regulatory proteins or micro RNAs of RNA sequences or structures located in untranslated regions of the transcript (Gebauer and Hentze, 2004). In plants, the importance of translational control has been reported during developmental processes such as flowering (Jiao and Meyerowitz, 2010), pollen tube germination (Hofmann, 2014), seed germination (Basbouss-Serhal et al., 2015), and abiotic stresses such as hypoxia (Branco-Price et al., 2008; Mustroph et al., 2009; Juntawong et al., 2014), drought (Kawaguchi et al., 2004), heat shock (Merret *et al*., 2017; Zhang et al., 2017), low temperature (Juntawong *et al*., 2013), light-dark transitions (Juntawong and Bailey-Serres, 2012; Missra et al., 2015), phosphate starvation (Bazin et al., 2017), and biotic stress (Meteignier et al., 2017; Xu et al., 2017).

It is interesting to note that both types of control could be occurring at the same time. For example, maize seedlings respond to hypoxia globally, reducing translation along with efficiently translating specific mRNAs such as alcohol dehydrogenase-1 (Adh1), a key enzyme to establish fermentative metabolism. The levels of Adh1 mRNA are low in roots in aerobiosis; however, hypoxia stimulates its transcription and translation (Bailey-Serres, 1999). 

Notably, in plants of the *Leguminosae* family, in which a symbiotic relationship with rhizobia is established, it has been shown that translation was not globally affected after rhizobia infection; instead, specific mRNAs coding for Nod factor receptors are selectively translated (Reynoso et al., 2013). Studies in the symbiosis between *Medicago truncatula* (*M. truncatula*) and *Sinorhizobium meliloti* (*S. meliloti*) showed that gene expression is strongly reprogramed at the translational level during the symbiotic interaction progression; moreover, identifying regulation cell type-specific of some mRNAs coding for hundreds of proteins and long noncoding RNAs (Traubenik et al., 2020). 

In the following sections of the review, we discuss and interrelate the methodologies for the analysis of polysome-associated mRNAs at the genome-scale to study the translational control and its importance in disentangling the nuances of the complex relationship between the legume plant and its symbiont.

## Translatome profiling: Methodologies for mRNA translation analysis at the genome-scale

As the relevance of the translational control of gene expression became evident, technologies were developed to analyze the translation of mRNAs on a genomic scale and at high resolution. Since mRNAs with higher translational activity are associated with more ribosomes, these techniques aim to determine the degree of association of each mRNA with polysomes to estimate its degree or level of translation. Polysome profiling (or polysome sequencing; Poly-seq) and Ribosome profiling (also known as ribosome footprinting or ribosome sequencing; Ribo-seq) are the two main methods for mRNA translation analysis, i.e. they are specific forms of RNA-seq analysis in which the subpopulations of mRNA directly bound to ribosomes are used (Chassé et al., 2017; Juntawong *et al*., 2018). The most relevant difference between them is that Poly-seq utilizes polysomal RNA for sequencing, whereas Ribo-seq is a footprinting approach restricted to sequencing RNA fragments protected by ribosomes (Ingolia et al., 2009; Aspden et al., 2014; Eastman *et al*., 2018). On the other hand, a common aspect of both profiling methodologies is that, in parallel, changes in total RNA levels are usually measured to study the relationship between transcription and translation impairment under different conditions and to distinguish between genes subjected to transcriptional and translational control (King and Gerber, 2016). 

**Figure 2 -:**
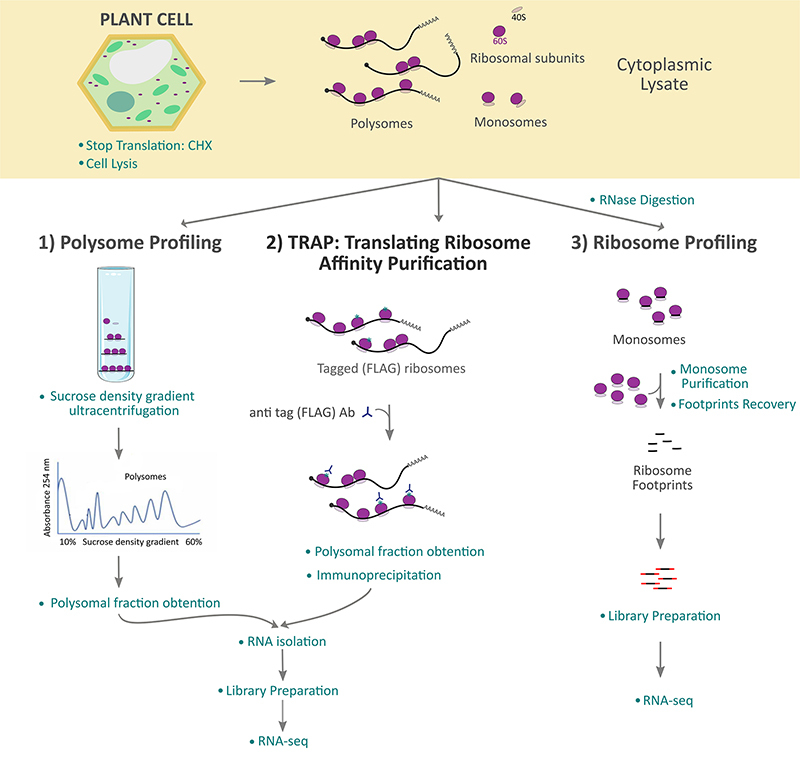
Schematic overview of the main tools for mRNA translation analysis at the genome-scale in the plant field. After plant tissue collection and flash freezing, translation is halted with cycloheximide (CHX), and a cell lysate is obtained. The cytoplasmic lysate containing polysomes, monosomes, and ribosomal subunits could be used for further ribosome-associated RNA isolation following different techniques: **1)** Polysome Profiling is a technique that separates actively translating mRNAs using sucrose density gradient ultracentrifugation. Once the polysome fraction is obtained, Trizol RNA isolation and conversion into cDNA libraries are performed for RNA sequencing (RNA-seq). **2)** TRAP (Translating Ribosome Affinity Purification) is based on the epitope tagging of a ribosomal protein for the immunopurification of ribosome-mRNA complexes. Cytoplasmic lysate from transgenic plants with ribosomal protein tagging undergoes immunoprecipitation with anti-tag antibodies to isolate the polysomal fraction, followed by Trizol RNA isolation and conversion into cDNA libraries for performing RNA-seq. **3)** Ribosome Profiling is a technique that treats cytoplasmic lysates with RNA-digesting enzymes to degrade the ribosome-unprotected mRNAs. After RNase digestion, monosomes are isolated, and ribosome-protected mRNA fragments (footprints) are purified and converted into cDNA libraries for RNA-seq.

Polysome profiling is a classical technique that uses sucrose density gradient centrifugation to separate actively translating mRNAs according to their density, determined by the number of ribosomes bound, followed by the deep sequencing of the mRNAs associated with the polysomal fraction (Chassé et al., 2017) (Figure 2). Unbound (ribosome-free) mRNAs and monosomal mRNAs are thought to be less actively translated than polysomal mRNAs. While the method is simple and robust, the approach is labor-intensive: several fractions from the gradient must be analyzed to measure translation efficiency sensitively (King and Gerber, 2016). Also, this methodology has the disadvantage that the polysomal fraction obtained may be contaminated with other high molecular weight ribonucleoprotein complexes (mRNPs), i.e. with a high sedimentation coefficient, such as pseudo-polysomes, P (processing) bodies, and storage granules among other mRNPs (Halbeisen and Gerber, 2009) that co-sediment with polysomes during ultracentrifugation (Thermann and Hentze, 2007). 

An alternative method to the sucrose density gradient approach for obtaining the polysomal fraction is immunoprecipitating the ribosomes enriched for polysome-associated mRNAs (Figure 2). In this technique developed in *Arabidopsis thaliana*, called TRAP for “Translating Ribosome Affinity Purification”, a small epitope tag is added to an exposed terminus of a ribosomal protein that has no detectable effect on ribosome function or polysome formation (Zanetti et al., 2005; Bailey-Serres, 2013). Specifically, the protein and tag used for this matter are the large subunit ribosomal protein 18 (RPL18) and the FLAG epitope tag (Zanetti *et al*., 2005). While this technique comprises the construction of transgenic plants that express FLAG-RPL18, which can be a challenge depending on the species under study, it is relatively simple to perform, not requiring specialized equipment, it is robust, and it can be adapted to immunopurify ribosome-associated mRNAs from specific cell populations (Mustroph et al., 2009). In both alternative methodologies for polysomal fraction obtention (sucrose gradient ultracentrifugation and TRAP), it is essential to immobilize the polysomes by means of treatment with cycloheximide (or alternatively with harringtonine, 5’-Guanylyl imidodiphosphate, chloramphenicol or flash freezing), an antibiotic that diffuses rapidly in the cells, stopping the cytosolic ribosomes in the initiation and elongation stages of translation (Obrig et al., 1971), and therefore avoiding polysomal run-off.

When a ribosome reads a mRNA template to guide protein synthesis, it encloses a region of approximately 30 nucleotides of the mRNA under translation; therefore, that region is protected from digestion by nucleases. The Ribosome Profiling methodology, described by Ingolia and collaborators, uses deep sequencing of those ribosome-protected mRNA fragments to determine the position of ribosomes on mRNA sequences at sub-codon resolution. Precisely, the approach consists of the immobilization of the ribosomes in the mRNAs by treatment with cycloheximide followed by the digestion of the mRNA with nucleases (step called RNase protection assay) that degrade the sequences not protected by the ribosome, leading to the formation of monosomes (Ingolia *et al*., 2009). Then, the monosomes are isolated (by different means such as sucrose gradients or cushions, commercial columns, or through the affinity purification of tagged-ribosomes), and the ribosome-protected mRNA fragments or footprints are purified, converted to cDNA libraries, and deep-sequenced to identify the precise positions occupied by the ribosomes on each mRNA (Figure 2). This analysis is based on the approximation that the density of ribosomes bound to an mRNA correlates with the level of synthesis of the corresponding protein, assuming that elongation rates are constant (Ingolia *et al*., 2012). By aligning the footprint sequences against the corresponding mRNA database and calculating the expression levels from the count of aligned sequences, the estimated levels of translation of each mRNA can be known. The abundance of footprints in the sequencing reports gives information on the expression level of each gene. Furthermore, it reveals the exact regions of the transcriptome that are being translated (Ingolia *et al*., 2012).

The profiles obtained with this technique contain various types of information about translation *in vivo* (Ingolia et al., 2009; Ingolia, 2016) as the discovery of new regulatory mechanisms. The presence of footprints on a region of RNA strongly suggests that it is translated, and although ribosomal footprints are expected to map to coding regions, the study by Ingolia *et al*. (2009) in yeasts reported that a small fraction of footprints (1.2%) map to non-coding regions. This way, Ribo-seq has revealed translational regulation invisible to normal mRNA measurements (Ingolia, 2016). Moreover, since ribosomal footprints are precise enough to observe the triplet periodicity, the identification of the reading frame, non-canonical initiation codons, and stop codons is possible (Ingolia *et al*., 2009; Ingolia, 2016).

Since both main approaches for translatome assessment, Poly-seq and Ribo-seq, exhibit technical limitations, have distinct strengths over each other, and generate complementary information, parallel evaluation of the translatome with these two approaches can generate a complete picture of how translational control determines protein output (Jin and Xiao, 2018). Polysome profiling cannot provide information on ribosome positioning within the transcript; it only estimates density (number of ribosomes per mRNA) and occupancy (number of mRNAs with and without ribosomes) (Jin and Xiao, 2018). Instead, deep sequencing of ribosome footprints provides information about ribosome positions as well as measuring expression quantitatively (Ingolia et al., 2012). However, a great advantage of the Poly-seq method is that it does not require gene manipulation of the tissue of interest.

## Contribution of the analysis of the translatome to the understanding of legume-rhizobia symbiosis

Multiple tiers of post-transcriptional regulation of gene expression, including translational control, are relevant in legume-rhizobia endosymbiosis (Reynoso et al., 2013; Traubenik et al., 2020). As was observed in plant-pathogen interactions, translatome analysis of the root-nodule symbiosis showed a limited correlation between transcriptional and translational changes, identifying genes with homodirectional (coupled variations between transcriptome and translatome; Tebaldi *et al*., 2012) and heterodirectional (uncoupled variations between transcriptome and translatome; Tebaldi *et al*., 2012) changes at both levels (Traubenik *et al*., 2020; Zanetti et al., 2020). Nonetheless, studies related to the characterization of translational regulation in nitrogen-fixing symbiosis are yet scarce, and this field of study is still in its infancy. As an example, a work from our group comprising a transcriptomic and translatomic analysis of the roots of nodulated and water-restricted plants, i.e. roots subjected to both biotic and abiotic signaling, showed that some members of the thioredoxin and glutaredoxin systems were regulated exclusively at the translational level denoting the importance of these enzymes for having a specific role in nodulated plants subjected to water deficit (Sainz et al., 2022a ). 

In an original study where the translatome of the roots of *M. truncatula* during the symbiotic interaction with its microsymbiont was analyzed through polysome purification, both by conventional sucrose gradient ultracentrifugation and TRAP, Reynoso et al. (2013), showed that some protein-coding mRNAs involved in nodulation suffer significant heterodirectional variation, with the changes occurring mainly at the translatomic level. Some of the translationally up-regulated mRNAs include receptor-like kinases (*Nod Factor Perception, NFP; does not make infection, DM12*), transcription factors of the GRAS family (Nodulation signaling Pathway 1 and 2, NSP1, NSP2), and nuclear factor Y (NF-Y) family (NF-YA1 and NF-YC1), among others, all of which are required for the successful formation of nitrogen-fixing nodules (Reynoso *et al*., 2013). Notably, these authors also showed that not only mRNAs but also micro RNAs (miRNAs) such as miR169 (known to be involved in nodule development; Combier et al., 2006) and miR172 are also subject to differential recruitment to polysomes, evidencing that differential translation of mRNAs and differential association of miRNAs to polysomes significantly contributes to the regulation of gene expression during the root nodule symbiotic pathway (Reynoso *et al*., 2013). 

Recently, the work of Traubenik et al. (2020) went further in the parse of translational regulation during the early stages of legume-rhizobia symbiosis, exploring the translational changes not only in a genome-wide scale through a TRAP-seq analysis but also in a root-specific cell type manner. These authors revealed, once again, a poor correlation between transcriptional and translational changes and identified many protein-coding and long non-coding RNAs (lncRNAs; non-coding transcripts of more than 200 nucleotides with limited coding potential). Moreover, the variations at the level of polysome association of these mRNAs and lncRNAs were found to be strongly influenced by the cellular context, i.e. their translational regulation occurs specifically in some root cell types. Thus, this work highlights not only the limited correlation between transcriptional and translational changes during the early stages of *M. truncatula* - *S. meliloti* interaction, which involves reprogramming root cells but also that the selective translation of many coding RNAs and selective association to the translational machinery of noncoding RNAs is important in those cells engaged in symbiosis. This information is novel in the symbiosis context, although the lack of correlation between the two previously mentioned regulatory levels had already been reported for other plant-microbe (pathogen) interactions.

## Simultaneous transcriptomic analysis of interacting symbionts

Symbiotic interactions, such as those between leguminous plants and rhizobia, involve intricate gene expression networks. Dual RNA-seq, i.e. the simultaneous sequencing of multi-species RNA isolated from the same biological sample, emerges as a valuable tool for exploring these complex relationships. Although initially developed for studying host-pathogen systems (Westermann et al., 2012), dual RNA-seq can also be applied to analyze RNA-seq data from the plant-rhizobia symbiotic pair simultaneously, enabling the investigation of coordinated gene expression patterns during symbiosis initiation, establishment, and responses to environmental cues (Mergaert et al., 2020). Moreover, integrating dual RNA-seq with strategies for studying the translatome can help uncover translation regulatory mechanisms within these partnerships. 

**Figure 3 -:**
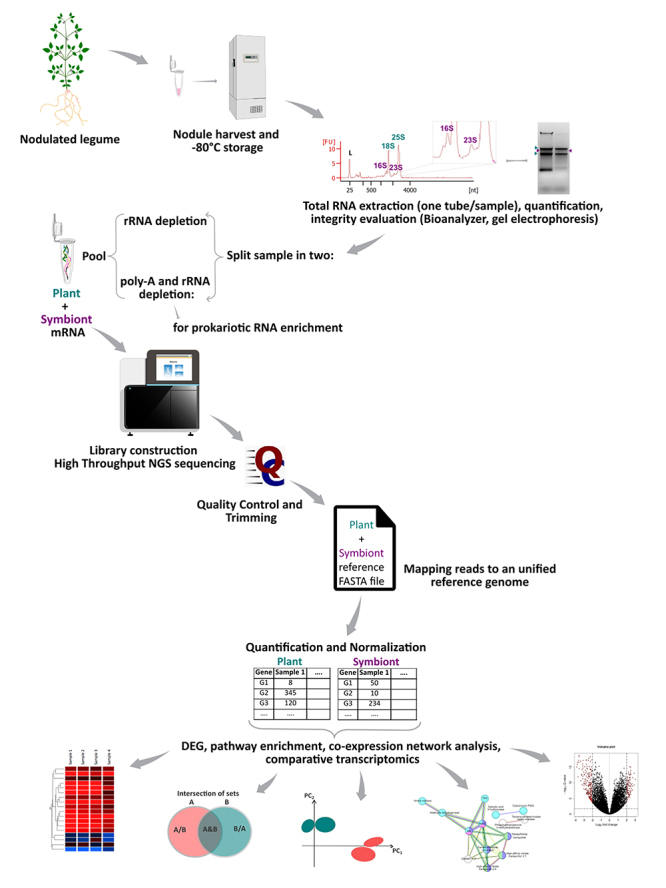
Schematic representation of the Dual-Seq protocol for plant-rhizobium symbiotic samples, including wet lab and the basic data analysis workflow. Fresh symbiotic nodules are detached from the plant roots, flash-frozen, and stored at -80 °C until used. Long storage periods are detrimental both for eukaryotic and prokaryotic RNAs but mainly for the prokaryotic ones. After total RNA extraction, quantification and integrity evaluation of the samples is performed. In the ribosomal RNA (rRNA) depletion step, each sample is split in two to perform prokaryotic RNA enrichment along with the rRNA depletion. Once the two previously split samples are pooled, cDNA sequencing libraries are made, followed by high throughput NGS sequencing. The basic data analysis workflow comprises visual quality inspection, followed by adapter and low-quality bases trimming. Then, reads are mapped to an unified (i.e. plant + symbiont) reference genome, followed by per gene read quantification and normalization. Differential gene expression (DEG) analysis, pathway enrichment analysis, co-expression network analysis, and comparative transcriptomic, among others, can be performed to interpret the data.

A typical dual RNA-seq experiment involves several steps: (i) sample preparation, (ii) isolation of eukaryotic+prokaryotic mRNA, (iii) library preparation, (iv) next-generation sequencing (NGS) and (v) data analysis (Marsh et al., 2018) (Figure 3). Additionally, applying strategies for polysome/ribosome-associated mRNA isolation during step (ii) makes conducting dual RNA-seq for both transcriptomic and translatomic studies possible. Dual RNA-seq has been used for transcriptome analysis in various symbiotic pairs (e.g., *M. truncatula - S. meliloti, Glycine max - Sinorhizobium,* and *Phaseolus vulgaris - Paraburkholderia phymatum*; Sauviac et al., 2022, Roux et al., 2014, Cui et al., 2021, Bellés-Sancho et al., 2021, respectively). As an example, the dual RNA-seq in *M. truncatula - S. meliloti* nodules undergoing senescence, either naturally or due to environmental triggers (nitrate treatment or salt stress), revealed numerous differentially regulated plant and bacterial genes associated with nodule senescence (Sauviac *et al*., 2022). Notably, core nodule senescence plant genes (such as MtNAC969 and MtS40) were found to negatively regulate the transition from nitrogen fixation to senescence, while the overexpression of a cytokinin biosynthesis gene, known for its role in leaf senescence inhibition, appeared to promote this transition in nodules. 

On the other hand, the study of the translatome in symbiotic interactions remains underexplored. Proper sample preparation, encompassing collection, lysis, RNA isolation, and quantification, is crucial for obtaining accurate and unbiased results. However, it presents significant challenges. Special care should be taken during RNA isolation to prevent transcriptional alterations and/or RNA degradation during handling (Wolf et al., 2018), while RNA isolation methods should be optimized to account for the distinct characteristics of plant and bacterial cells, including cell walls. Added to this well-known RNA handling issue, dual RNA extraction could undergo a representation issue. RNA content can vary significantly among species, resulting in large differences in RNA abundance (Westermann et al., 2012). Indeed, bacteria in symbiosis typically yield considerably less RNA than their host plant cells (e.g. Roux et al., 2014; Bellés-Sancho et al., 2021; Cui et al., 2021; Sainz et al., 2022b ; Sauviac et al., 2022), sometimes differing by several orders of magnitude. This aspect underscores one of the main challenges in dual RNA-seq: achieving high-quality RNA extraction that faithfully represents both organisms, thereby minimizing bias (Chung *et al*., 2021). In terms of RNA quantification and integrity assessment, the use of Bioanalyzer, the gold standard instrument for these purposes, also encounters challenges. The presence of ribosomal subunits of different sizes in the sample can lead to errors in the automatic calculation of the RNA Integrity Number (RIN), resulting in an inaccurate assessment of RNA integrity and a misleading perception of degradation (Sainz *et al*., 2022b). In those cases, relying on Qubit for quantification and agarose gel electrophoresis for integrity assessment emerge as the options of choice. 

A persistent issue in library preparation for dual RNA-seq experiments is the incomplete removal of ribosomal RNA (rRNA), which is the most abundant RNA type in cells (>95%). Unlike traditional RNA-seq in eukaryotes, poly(A) cannot be used for mRNA enrichment in dual RNA-seq because prokaryotes lack this distinctive feature (Giannoukos et al., 2012). Instead, selective removal kits like Illumina Ribo-Zero or NEBNext are used for rRNA depletion, with any remaining rRNA reads removed *in silico* based on rRNA sequence databases. Conversely, the rRNA depletion step can serve as a strategy to enrich the sample in prokaryotic mRNA. The sample can be split into two portions, with one undergoing polyA depletion alongside rRNA depletion, thereby enriching prokaryotic RNA (i.e. without polyA). The other half only undergoes rRNA depletion. Upon pooling both halves, the prokaryotic fraction result enriched, changing the eukaryotic/prokaryotic RNA ratio (Chung *et al*., 2021).

Regarding NGS, transcriptome coverage is a central aspect. Dual RNA-seq demands a high coverage, which becomes even more critical as the eukaryotic/prokaryotic RNA ratio increases. Nevertheless, reliable results have been obtained even with fewer than 40 million non-ribosomal reads (e.g., Li et al., 2022). Common Illumina read lengths typically range from 100 to 200 base pairs, although longer reads offer more reliable genome localization. Paired-end sequencing, which sequences fragments from both ends, enhances data quality and is particularly useful for avoiding multi-mapping and detecting novel transcript variants (Wolf et al., 2018). More recently, long-read sequencing technologies, such as PacBio and Oxford Nanopore, have proven the capacity to span entire transcript lengths, enhancing the precision of isoform structure determination. In addition, Nanopore has the capability to sequence RNA directly, eliminating the need for cDNA conversion. In contrast, conventional technologies like Illumina and Ion Torrent tend to offer higher levels of precision at lower cost, usually becoming the sequencing method of choice (Marsh et al., 2018).

Once raw NGS reads are obtained, data processing involves (i) quality control and trimming, (ii) mapping the reads to reference genomes/transcriptomes, (iii) quantifying the number of reads per gene, and (iv) identifying differentially expressed genes (DEGs; (Marsh et al., 2018) (Figure 3). Several specialized bioinformatics pipelines have been developed to handle RNA-seq data, although they often require programming skills. This way, quality control and trimming are typically performed using tools like FastQC (Andrews, 2010) and Trimmomatic (Bolger et al., 2014), while read mapping is accomplished using mapping tools like Bowtie2 (Langmead and Salzberg, 2012) or HISAT2 (Kim et al., 2019). Subsequently, tools such as featureCounts (Liao et al., 2014) or Salmon (Patro et al., 2017) are utilized to calculate the number of reads per gene, while DEGs can be identified using diverse statistical methods, many of which are available in R/Bioconductor (R Core Team, 2021; Gentleman et al., 2004). Alternatively, user-friendly tools with graphical interfaces, such as RNA CoMPASS (Xu et al., 2014) and the Galaxy platform (Afgan et al., 2018), offer a more accessible data analysis option, albeit with some trade-offs in terms of control and flexibility (Wolf et al., 2018). The best practices for dual RNA-seq data analysis can be reviewed in (Chung *et al*., 2021).

In dual RNA-seq experiments, RNAs from interacting species are distinguished during the mapping process, either through sequential mapping to the genomes of both species or by mapping to a single concatenated genome (Marsh et al., 2018). The latter approach is often preferred due to its computational efficiency, as it requires mapping only once, allowing the mapping tool to determine the most appropriate genome match for each read (Li et al., 2022). Data interpretation is a critical phase involving clustering, enrichment analysis, and gene classification based on functions and associations with the trait of interest (Figure 3). Clustering methods facilitate the grouping of genes with similar expression profiles, often revealing shared functions or pathways, while gene co-expression networks allow the identification of genes with highly similar expression patterns to one another. Enrichment analysis, typically employing Gene Ontology (The Gene Ontology Consortium, 2023) or KEGG (Kanehisa et al., 2009) terms, provides a comprehensive overview of pathways or functions enriched with DEGs. Additionally, when polysome/ribosome-associated RNA sequencing is performed alongside total mRNA sequencing, it becomes feasible to identify genes involved in translational, transcriptional, or mixed (i.e. transcriptional + translational) regulation, through a comprehensive analysis strategy that enables the discrimination of overlapping DEGs across different regulatory levels.

The increasing emphasis on open science and open data underscores the importance of sharing not only raw sequencing data but also metadata and processed expression data. Consequently, there is a growing need to develop databases with curated data. Among these, databases dedicated to dual RNA-seq, like DualSeqDB (Macho Rendón et al., 2021), aim to facilitate the exploration of gene expression changes during infection at both the host and pathogen levels. As the field progresses, specialized databases tailored for symbiotic interactions are expected to emerge in the near future.
